# Case Reports of Heroin Injection Site Necrosis: A Novel Antecedent of Nicolau Syndrome

**DOI:** 10.7759/cureus.29235

**Published:** 2022-09-16

**Authors:** Brandon Rose, Thomas G Powell, Michelle Jones, Shawn A Chillag, Suzanne Kemper

**Affiliations:** 1 Internal Medicine, Baptist Health Medical Group, Kentucky, USA; 2 Transitional Year, Conway Medical Center, Conway, USA; 3 Pulmonary-Critical Care, Prisma Health, Greenville, USA; 4 Internal Medicine, West Virginia University School of Medicine, Charleston, USA; 5 Outcomes Research, Charleston Area Medical Center Health Education and Research Institute, Charleston, USA

**Keywords:** opioid-abuse, drug abuse, necrotizing fasciitis, livedoid dermatitis, nicolau syndrome, adulterated heroin, heroin injection complication, skin necrosis, clinical case report

## Abstract

Heroin injection-site necrosis (HISN) is a novel and poorly understood complication of intravenous drug abuse (IVDA). We present three cases of HISN that were evaluated and treated in Charleston, West Virginia, in 2019 and 2020. The documented cases show similarities involving patient care, follow-up, clinical progression, patient demographic, and dermatologic sequelae. We discuss these similarities, provide clinical recommendations, review proposed etiologies of HISN, and introduce Nicolau syndrome as a potential mechanism.

## Introduction

Opioid use in the United States has been identified by multiple medical organizations as an epidemic, with heroin being the most abused by injection [1]. Numerous well-known complications are associated with heroin injection, ranging from endocarditis to overdose [1]. A relatively novel complication is a necrotic skin condition dubbed heroin injection-site necrosis (HISN). The total number of cases is unknown, as HISN is not frequently reported in the literature. Here, we present three cases of HISN and discuss their similarities to previous reports, provide clinical recommendations, and review proposed etiologies of HISN. While various reports have identified multiple etiologies for HISN, no definitive cause has been identified [2,3]. Multiple antecedents coupled with commonalities in the clinical presentation and progression may help elucidate a shared mechanism.

## Case presentation

Case 1

**Figure 1:**
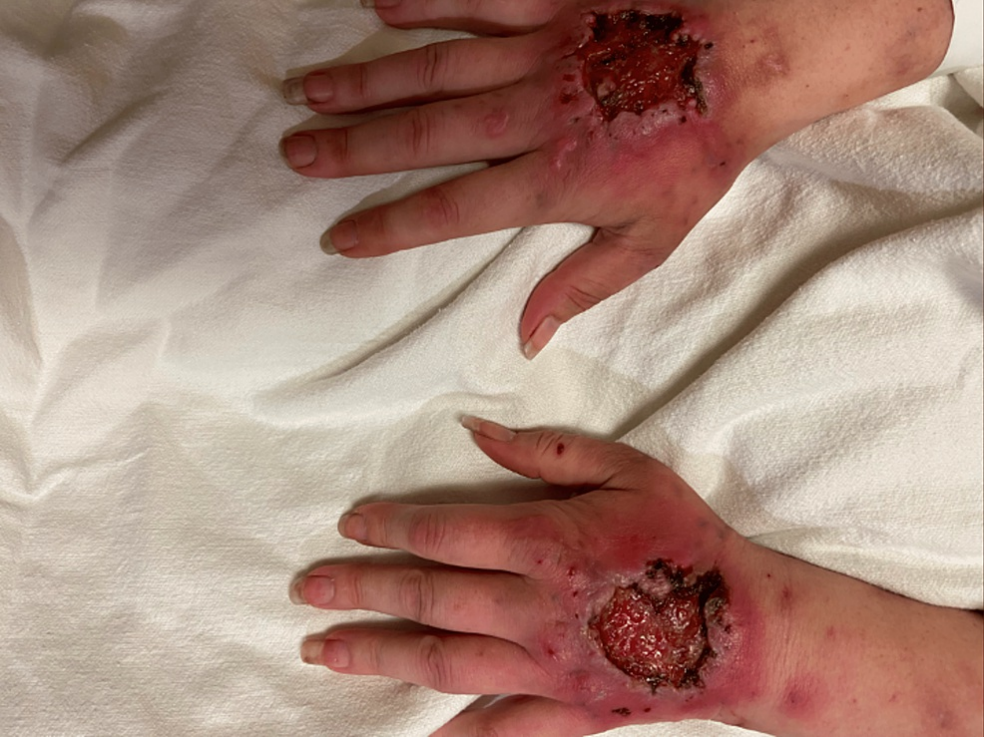
Heroin-induced skin necrosis of the bilateral dorsal hands with erythematous ulcerations

This is a 31-year-old woman with a history of intravenous drug abuse (IVDA) with wounds on the dorsal surface of both hands. These lesions, where she previously injected heroin, progressed over several months. They began as erythema with associated pain and advanced to solitary ulcers. She denied the use of any other substances and was certain that her dealer used fiber supplements as a bulking agent. On presentation, there was marked erythema and significant tenderness around the lesions as seen in Figure 1. The remainder of her physical exam was normal. She was hemodynamically stable and afebrile. She denied any primary psychiatric history, self-harming practices, or systemic autoimmune disease. Wound culture identified a methicillin-resistant Staphylococcus aureus (MRSA) infection. She underwent bilateral debridement and grafting of the wounds and was treated with intravenous (IV) antibiotics. Following treatment, she did not follow up and has not been seen since hospital discharge. No pathology was obtained for this patient.

Case 2

**Figure 2:**
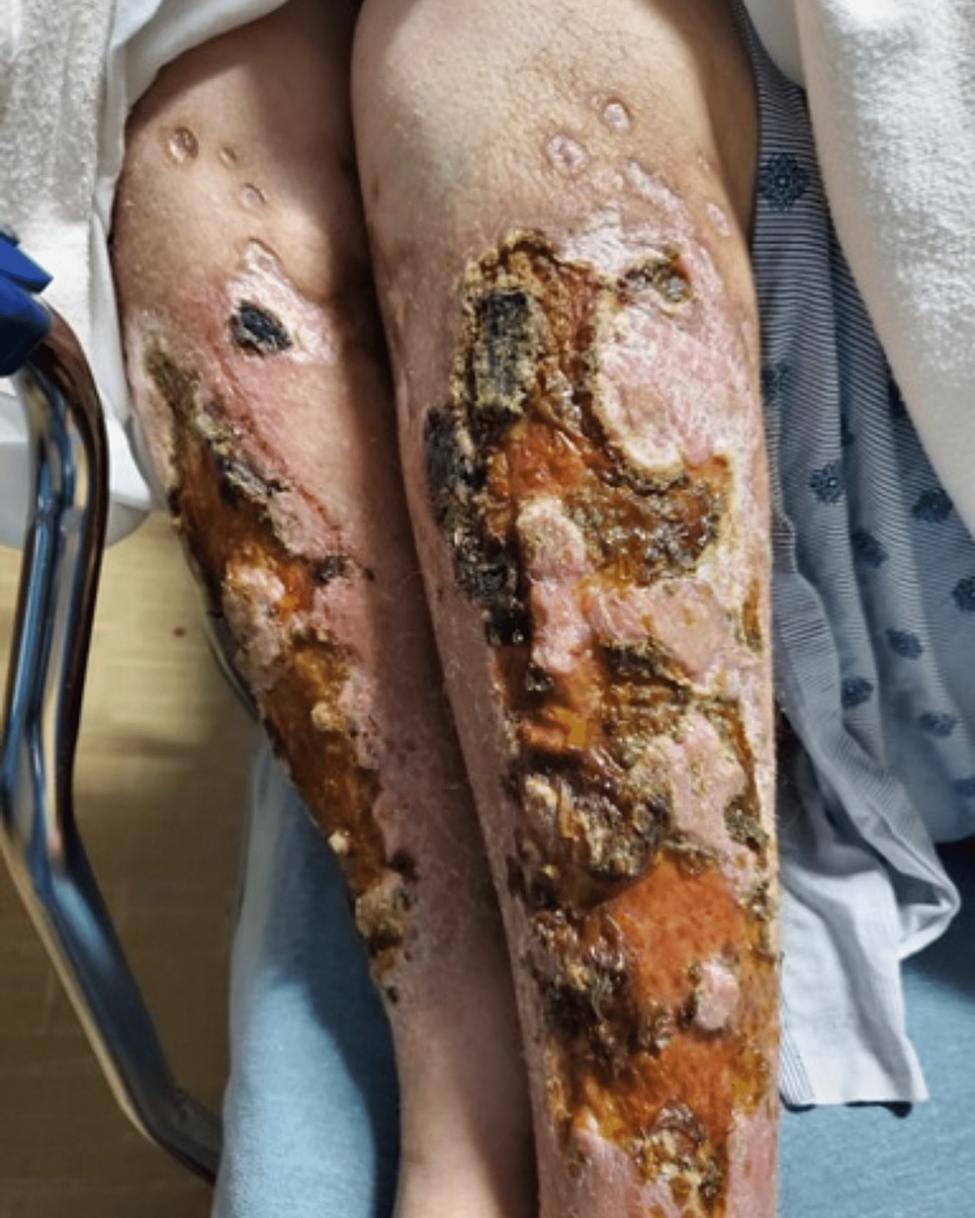
Heroin-induced skin necrosis on ventral bilateral lower extremities with coalescing necrotic ulcers and necrotic scaling

This is a 44-year-old woman with a history of IVDA with wounds on the ventral aspects of her upper and lower extremities. These lesions, where she previously injected heroin, progressed over weeks. They began with erythema and associated pain. Over time, ulcerations developed, which began to coalesce with necrotic tissue and large alternating black and brown scales as seen in Figure 2. The remainder of her physical exam was normal. She was hemodynamically stable and afebrile. She denied any primary psychiatric history, self-harming practices, or systemic autoimmune disease. She was started on IV antibiotics while in the emergency department and transferred to the medical floor for further workup and observation. Shortly after beginning treatment, she left against medical advice (AMA). She has not followed up since that time. No pathology or microbiology results were obtained for this patient.

Case 3

**Figure 3:**
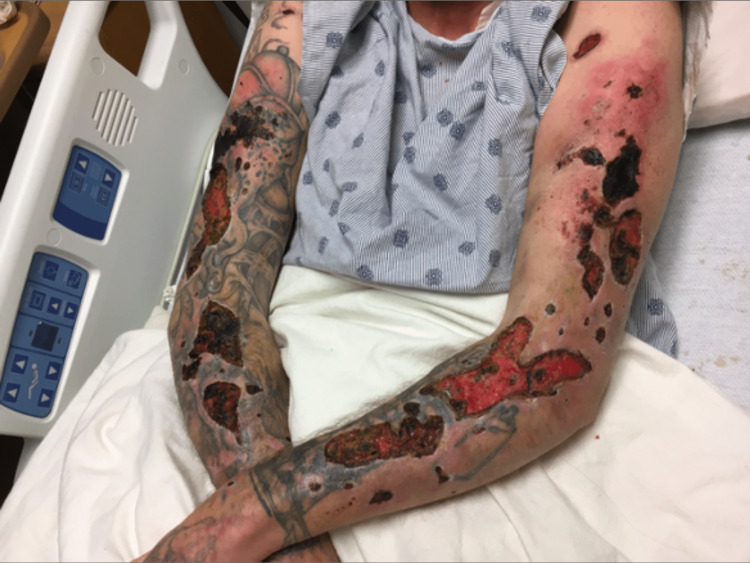
Heroin-induced skin necrosis on the dorsal bilateral upper extremities with scattered necrotic ulcers and necrotic scaling

This is a 31-year-old man with a history of IVDA with multiple wounds on the dorsal aspect of his bilateral upper extremities. These lesions began as erythema and pain at sites of “gray heroin” injection, which progressed to scattered ulcerations. On discovering the lesions, he attempted a home debridement with a knife and pair of scissors. At presentation, there was marked erythema around most of the ulcers and multiple, contained, black necrotic tissue as seen in Figure 3. The remainder of his physical exam was normal. He was hemodynamically stable and afebrile. He denied any primary psychiatric history, self-harming practices, or systemic autoimmune disease. Tissue debridement showed MRSA infection and necrosis without vasculitis. He received IV antibiotic therapy and underwent a skin grafting procedure. He was healing well at discharge.

## Discussion

In previously reported cases of HISN, various causes have been implicated. These include bulking agents, novel opioids, and bacterial infections. Bulking agents are supplements added to increase the volume of heroin and have unknown effects on the human body [2]. Houck et al. reported the use of “Rizzy,” a powdered flower preservative, as filler material [2]. Our first case demonstrates how a fiber supplement was the suspected bulking agent. Another proposed mechanism of HISN is the use of novel opioids, such as desomorphine, coined krokodil, which produces devastating skin necrosis. Though discovered in Russia, its presence has been recently reported in the United States [3]. Our third case involved the reported use of “gray heroin,” which may suggest a previously identified opioid mixture termed “gray death” [4]. Finally, the two cases where wound cultures were obtained grew MRSA but without signs of necrotizing soft tissue infection [5].

Although previous reports have suggested various etiologies of HISN, the clinical presentation and course remain similar, suggesting a common pathophysiologic mechanism. We suspect Nicolau syndrome as a likely explanation. This syndrome commonly results from the direct arterial injection of non-steroidal anti-inflammatory drugs, penicillin, and vaccines leading to skin necrosis with a similar clinical course as seen in HISN [6,7]. We presume that people who engage in IVDA are likely to experience this phenomenon due to inadvertent arterial injection.

Our cases characterize similar clinical findings and patient characteristics found in previous reports of HISN. These patients typically present late in disease progression and are surprisingly hemodynamically stable and afebrile on presentation [2]. Patients afflicted with HISN are typically Caucasian, between the ages of 20 and 40, and equally male and female [2,3]. Haskin et al. describe HISN dermatologic findings as pain and erythema at injection sites followed by ulceration and necrotic scaling [3]. This evolution appears relatively standard amongst reported cases and typically involves the extremities [2,3]. All our cases began as erythema and pain at previous sites of heroin injection with the subsequent development of ulcers. One case demonstrated isolated ulcers with no necrotic scaling, and the other two exhibited ulcers with subsequent scale development. We postulate that HISN dermatologic sequelae begin as ulcerations, progressing to scaling necrosis typically involving the extremities. Two cases were lost to follow-up, limiting our ability to map disease progression and resolution. Further research into the clinical commonalities of HISN can aid in disease recognition and improve clinical results.

Before diagnosing HISN, necrotizing fasciitis (NF) should be ruled out. HISN, which can be superinfected with bacteria, may be initially difficult to distinguish from NF, which is a rapidly progressive primary infection. Furthermore, organisms cultured in patients with HISN, notably Staphylococcus species and Clostridium species, are known causes of NF [8,9]. The lack of fever, hemodynamic instability, and subcutaneous emphysema suggest HISN rather than NF. A white blood cell count <15,000 cells/mm3 and serum sodium greater than 135 mmol/L have a negative predictive value of 99% for necrotizing soft tissue infections [5]. Finally, lesion evolution over weeks would suggest HISN while development within hours suggests NF [5].

These cases share aspects with both pyoderma gangrenosum (PG) and dermatitis artefacta, which need to be considered to properly make the diagnosis of HISN. Our suggested clinical progression of HISN is similar to that of PG, both forms of noninfectious inflammatory dermatitis [10]. The absence of systemic inflammatory disease in our cases suggests HISN, as it is seen in nearly half of PG patients [10]. Also, PG characteristically presents with dense neutrophilic infiltrate and vasculitis, which our case with biopsy demonstrated necrosis without vasculitis [10]. Indeed, more investigation of the histological features of HISN is required to help differentiate it from PG and other ulcerative diseases. Finally, dermatitis artefacta is a psychocutaneous disorder, wherein the patient has a psychological need to manually induce lesions without proprietary benefit [11]. This disease is within a spectrum of self-infected dermatoses, which are notoriously difficult to diagnose [11]. Our cases lack psychiatric history aside from substance abuse disorder and the typical psychological stressors seen in dermatitis artefacta, making this disease less likely [11].

Obtaining a thorough drug use history is crucial in patients with HISN; for example, inquiring about the heroin type, filler material use, or change in primary source. Indeed, street heroin may not actually be heroin. One of our cases involved the use of a reported “gray heroin.” It is likely the substance was the poly-opioid termed “gray death” [4]. Reports of gray death were made in Ohio and Pennsylvania during the timeframe of our cases, and in West Virginia as recently as October 2021 [4,12]. Our toxicology analysis demonstrated a mixture of substances, which supports this theory [4]. Specific formulations of opioids are prevalent in a particular area at one time [13,14]. Geographic and chronological tracking within a population may assist clinicians in recording HISN incidence with specific types of opioids. These cases were shared with appropriate authorities as a potential public health problem. We recommend clinicians investigate the culprit substance in cases of HISN and follow local reporting protocols.

An identified obstacle when treating IVDA patients is the perceived undertreatment of pain [15]. These individuals commonly report their pain as undertreated, thus influencing their decision to leave AMA. One contributing factor may be the skepticism of clinicians using opioids in this demographic. HISN is an insufferable condition, and IVDA patients commonly adapt to a high opioid tolerance [1]. Clinicians need to consider this bias to ensure adequate pain management and minimization of AMA risk. We suspect the undertreatment of pain played a role in two of our patients leaving AMA.

## Conclusions

In conclusion, HISN is an ulcerative skin condition in people who inject heroin. Bacteria, filler agents, and novel opioids have been implicated as HISN etiologies; however, Nicolau syndrome may provide a better explanation. Our cases demonstrate similarities in clinical findings and patient characteristics when compared to previous reports of HISN. While both benefit from antibiotic therapy and debridement, the subacute nature of HISN and lack of hemodynamic instability help distinguish it from NF. It is vital to consider other etiologies of ulceration if HISN is suspected, such as PG or dermatitis artefacta. Clinicians must obtain a thorough drug use history in patients with HISN and investigate local forms of adulterated opioids for chronological understanding and local healthcare awareness. Finally, optimizing pain management may improve patient adherence and recovery.

## References

[1] Strain E (2022). Opioid use disorder: Epidemiology, pharmacology, clinical manifestations, course, screening, assessment, and diagnosis. https://www.uptodate.com/contents/opioid-use-disorder-epidemiology-pharmacology-clinical-manifestations-course-screening-assessment-and-diagnosis?search=addiction&topicRef=7807&source=see_link.

[2] Houck J, Ganti L (2019). A local epidemic of laced heroin causing skin necrosis. Cureus.

[3] Haskin A, Kim N, Aguh C (2016). A new drug with a nasty bite: a case of krokodil-induced skin necrosis in an intravenous drug user. JAAD Case Rep.

[4] (2022). Gray death crisis: new killer heroin drug cocktail. https://drugabuse.com/drugs/heroin/gray-death-crisis/.

[5] Hakkarainen TW, Kopari NM, Pham TN, Evans HL (2014). Necrotizing soft tissue infections: review and current concepts in treatment, systems of care, and outcomes. Curr Probl Surg.

[6] Senel E (2012). Nicolau syndrome as an avoidable complication. J Family Community Med.

[7] Nischal K, Basavaraj H, Swaroop M, Agrawal D, Sathyanarayana B, Umashankar N (2009). Nicolau syndrome: an iatrogenic cutaneous necrosis. J Cutan Aesthet Surg.

[8] Raza N, Dhital S, Espinoza VE (2021). Wound botulism in black tar heroin injecting users: a case series. J Investig Med High Impact Case Rep.

[9] Jackson KA, Bohm MK, Brooks JT (2018). Invasive methicillin-resistant Staphylococcus aureus infections among persons who inject drugs - six sites, 2005-2016. MMWR Morb Mortal Wkly Rep.

[10] George C, Deroide F, Rustin M (2019). Pyoderma gangrenosum - a guide to diagnosis and management. Clin Med (Lond).

[11] Saha A, Seth J, Gorai S, Bindal A (2015). Dermatitis artefacta: a review of five cases: a diagnostic and therapeutic challenge. Indian J Dermatol.

[12] (2022). Money, guns, and ‘gray death’ found inside Mingo County home. https://www.wsaz.com/2021/10/21/money-guns-gray-death-found-inside-mingo-county-home/.

[13] (2022). Notes from the field: acetyl fentanyl overdose fatalities - Rhode Island, March - May 2013. https://www.cdc.gov/mmwr/preview/mmwrhtml/mm6234a5.htm.

[14] Armenian P, Olson A, Anaya A, Kurtz A, Ruegner R, Gerona RR (2017). Fentanyl and a novel synthetic opioid U-47700 masquerading as street “Norco” in central California: a case report. Ann Emerg Med.

[15] Simon R, Snow R, Wakeman S (2020). Understanding why patients with substance use disorders leave the hospital against medical advice: A qualitative study. Subst Abus.

